# The coming era of proteomics-driven precision medicine

**DOI:** 10.1093/nsr/nwaf278

**Published:** 2025-07-14

**Authors:** Ying Jiang, Jian Wang, Aihua Sun, Hongxing Zhang, Xiaobo Yu, Weijie Qin, Wantao Ying, Yanchang Li, Cheng Chang, Xiaowen Wang, Linhai Xie, Wei Liu, Jialin Liu, Xiaomei Zhang, Qunjiao Yan, Yu Zou, Chuanping Zhao, Haofan Sun, Jian Zhang, Shicheng Su, Qiang Gao, Fuchu He

**Affiliations:** State Key Laboratory of Medical Proteomics, Research Unit of Proteomics Driven Cancer Precision Medicine (Chinese Academy of Medical Sciences), Beijing Proteome Research Center, National Center for Protein Sciences (Beijing), Academy of Military Medical Sciences, Beijing 102206, China; State Key Laboratory of Medical Proteomics, Research Unit of Proteomics Driven Cancer Precision Medicine (Chinese Academy of Medical Sciences), Beijing Proteome Research Center, National Center for Protein Sciences (Beijing), Academy of Military Medical Sciences, Beijing 102206, China; State Key Laboratory of Medical Proteomics, Research Unit of Proteomics Driven Cancer Precision Medicine (Chinese Academy of Medical Sciences), Beijing Proteome Research Center, National Center for Protein Sciences (Beijing), Academy of Military Medical Sciences, Beijing 102206, China; State Key Laboratory of Medical Proteomics, Research Unit of Proteomics Driven Cancer Precision Medicine (Chinese Academy of Medical Sciences), Beijing Proteome Research Center, National Center for Protein Sciences (Beijing), Academy of Military Medical Sciences, Beijing 102206, China; State Key Laboratory of Medical Proteomics, Research Unit of Proteomics Driven Cancer Precision Medicine (Chinese Academy of Medical Sciences), Beijing Proteome Research Center, National Center for Protein Sciences (Beijing), Academy of Military Medical Sciences, Beijing 102206, China; State Key Laboratory of Medical Proteomics, Research Unit of Proteomics Driven Cancer Precision Medicine (Chinese Academy of Medical Sciences), Beijing Proteome Research Center, National Center for Protein Sciences (Beijing), Academy of Military Medical Sciences, Beijing 102206, China; State Key Laboratory of Medical Proteomics, Research Unit of Proteomics Driven Cancer Precision Medicine (Chinese Academy of Medical Sciences), Beijing Proteome Research Center, National Center for Protein Sciences (Beijing), Academy of Military Medical Sciences, Beijing 102206, China; State Key Laboratory of Medical Proteomics, Research Unit of Proteomics Driven Cancer Precision Medicine (Chinese Academy of Medical Sciences), Beijing Proteome Research Center, National Center for Protein Sciences (Beijing), Academy of Military Medical Sciences, Beijing 102206, China; State Key Laboratory of Medical Proteomics, Research Unit of Proteomics Driven Cancer Precision Medicine (Chinese Academy of Medical Sciences), Beijing Proteome Research Center, National Center for Protein Sciences (Beijing), Academy of Military Medical Sciences, Beijing 102206, China; State Key Laboratory of Medical Proteomics, Research Unit of Proteomics Driven Cancer Precision Medicine (Chinese Academy of Medical Sciences), Beijing Proteome Research Center, National Center for Protein Sciences (Beijing), Academy of Military Medical Sciences, Beijing 102206, China; State Key Laboratory of Medical Proteomics, Research Unit of Proteomics Driven Cancer Precision Medicine (Chinese Academy of Medical Sciences), Beijing Proteome Research Center, National Center for Protein Sciences (Beijing), Academy of Military Medical Sciences, Beijing 102206, China; International Academy of Phronesis Medicine (Guangdong), Guangzhou 510000, China; Academy of Military Medical Sciences, Beijing 100039, China; State Key Laboratory of Medical Proteomics, Research Unit of Proteomics Driven Cancer Precision Medicine (Chinese Academy of Medical Sciences), Beijing Proteome Research Center, National Center for Protein Sciences (Beijing), Academy of Military Medical Sciences, Beijing 102206, China; State Key Laboratory of Medical Proteomics, Research Unit of Proteomics Driven Cancer Precision Medicine (Chinese Academy of Medical Sciences), Beijing Proteome Research Center, National Center for Protein Sciences (Beijing), Academy of Military Medical Sciences, Beijing 102206, China; Academy of Military Medical Sciences, Beijing 100039, China; State Key Laboratory of Medical Proteomics, Research Unit of Proteomics Driven Cancer Precision Medicine (Chinese Academy of Medical Sciences), Beijing Proteome Research Center, National Center for Protein Sciences (Beijing), Academy of Military Medical Sciences, Beijing 102206, China; State Key Laboratory of Medical Proteomics, Research Unit of Proteomics Driven Cancer Precision Medicine (Chinese Academy of Medical Sciences), Beijing Proteome Research Center, National Center for Protein Sciences (Beijing), Academy of Military Medical Sciences, Beijing 102206, China; State Key Laboratory of Medical Proteomics, Research Unit of Proteomics Driven Cancer Precision Medicine (Chinese Academy of Medical Sciences), Beijing Proteome Research Center, National Center for Protein Sciences (Beijing), Academy of Military Medical Sciences, Beijing 102206, China; State Key Laboratory of Medical Proteomics, Research Unit of Proteomics Driven Cancer Precision Medicine (Chinese Academy of Medical Sciences), Beijing Proteome Research Center, National Center for Protein Sciences (Beijing), Academy of Military Medical Sciences, Beijing 102206, China; State Key Laboratory of Medical Proteomics, Breast Tumor Center, Sun Yat-Sen Memorial Hospital, Sun Yat-Sen University, Guangzhou 510120, China; Key Laboratory of Carcinogenesis and Cancer Invasion (Ministry of Education), Liver Cancer Institute, Zhongshan Hospital, Fudan University, Shanghai 200032, China; State Key Laboratory of Medical Proteomics, Research Unit of Proteomics Driven Cancer Precision Medicine (Chinese Academy of Medical Sciences), Beijing Proteome Research Center, National Center for Protein Sciences (Beijing), Academy of Military Medical Sciences, Beijing 102206, China; International Academy of Phronesis Medicine (Guangdong), Guangzhou 510000, China

**Keywords:** proteomics, post-translational modifications, protein–protein interactions, spatial proteome, precision medicine, phronesis medicine

## Abstract

The concept of ‘proteomics-driven precision medicine’ highlighted in 2019 emphasized the potential of proteomics to transform precision medicine by offering deeper insights into dynamic biological processes. Since then, this field has seen remarkable advancements, interlinking with key pillars such as protein expression profiling, post-translational modifications, protein–protein interactions and spatial proteomics to transform healthcare. Technological progress has led to the creation of comprehensive reference maps of proteomes, identification of over 90% of human protein-coding genes, and detailed cellular and molecular landscapes within organs. Proteomics has significantly advanced health monitoring and disease surveillance by developing aging models, predicting disease risks with superior protein risk scores, identifying biomarkers for early conditions like dementia, and securing Food and Drug Administration (FDA) approval for multiple cancer biomarkers. By providing deeper insights into the proteome's complexity and dynamics, proteomics is revolutionizing our understanding, diagnosis and treatment of diseases, firmly establishing itself as a cornerstone of precision medicine.

## INTRODUCTION

The push for precision medicine is underscored by advancements in disease classification systems, crucial for standardizing how diseases and health conditions are categorized. These systems, especially the International Classification of Diseases (ICD) by the World Health Organization (WHO), have been fundamental in diagnosis and health management but often lack customization for individual patient needs. A significant innovation has been the development of an ontology-based mechanistic classification, which incorporates diverse scientific and clinical data to define disease endotypes [1]. This approach enhances the personalization of medical treatment, aligning closely with the goals of precision medicine and marking a significant shift in healthcare towards individualized strategies.

The rise of genomics and molecular biology has emphasized the significance of analyzing individual genetic profiles, facilitating advances in precision medicine. Genome-driven precision medicine (GDPM) has seen significant successes in treating inherited diseases and in oncology, particularly with targeted therapies like imatinib for chronic myeloid leukemia [2] and EGFR-tyrosine kinase inhibitors for lung cancer [3], which have dramatically improved patient outcomes. However, research, including studies from the UK Biobank, suggests that the genetic contribution to many diseases may be lower than expected, with many cancers exhibiting low heritability [4] (Fig. S1) and limited benefit from genomically targeted therapy [5]. This realization prompts a need to broaden our understanding of disease mechanisms beyond genetic factors to enhance treatment efficacy further.

**Figure 1.**
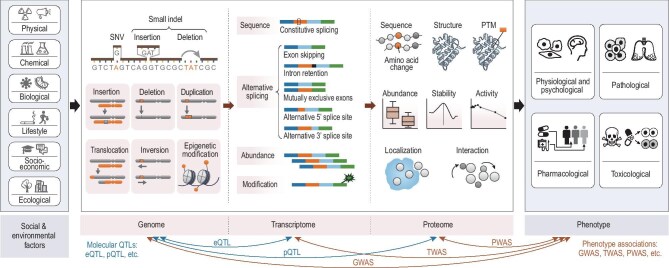
The pivotal role of the proteome, which has an impact on phenotype. The interplay between genetic and environmental factors defines the state of the transcriptome, proteome, metabolome etc., among which the proteome directly influences both health and disease phenotypes. Social and environmental factors include physical, chemical, biological, lifestyle, socioeconomic and ecological factors. The genome consists of functional elements that encode the blueprint of life, along with genetic variations that serve as the source of diversity. Genetic variations encompass single nucleotide variants (SNVs), small indels (insertion and deletion) and structural variations (SVs) such as large insertions, large deletions, duplication, translocation and inversion. Epigenetic modifications in the genome, such as DNA methylation, act as a dynamic regulatory layer that governs the activation or silencing of genes. The transcriptome encompasses the complete set of RNA transcripts produced by the genome. It includes coding mRNAs, non-coding RNAs, and their processing states, characterized by splicing diversity, transcript abundance and post-translational modifications (PTMs). The proteome includes all proteins with various sequences, structures, modifications, abundance, stability, activity, localization and interaction. Phenotypes shown here include physiological, psychological, pathological, pharmacological and toxicological traits. Expression quantitative trait loci (eQTLs) map genetic variants influencing RNA-level changes; pQTLs link genetic variants to variation of protein expression. Genome-wide association study (GWAS) identifies genetic variants linked to traits; transcriptome-wide association study (TWAS) prioritizes dysregulated genes whose expression correlate with phenotypes; proteome-wide association study (PWAS) maps protein-level alterations associated with disease or non-disease outcomes. Indel, Insertion and deletion.

**Figure 2.**
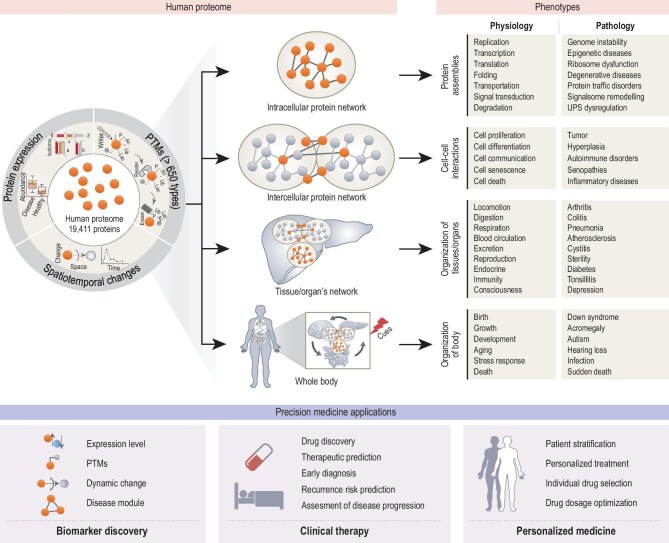
Human proteome to precision medicine: from molecular features to personalized health outcomes. The dynamic nature of protein expression and PTMs under diverse spatiotemporal conditions ultimately results in alterations to the PPI network. These interactions occur at multiple biological levels, including intracellular, intercellular, tissue/organ and individual scales. These hierarchical interactions yield physiological and pathological phenotypes. P, phosphorylation. Ac, acetylation; Ub, ubiquitination; Lac, lactylation.

The complex relationship between genetic and environmental factors is pivotal in shaping health outcomes. While genetic makeup provides the fundamental blueprint, it is the interaction with environmental influences that dictates the health trajectory of an individual [6]. This interaction affects the genome, transcriptome and proteome, highlighting the role of proteins, key executors of genetic information, in all biological processes. Proteomics, the large-scale/ome-wide view study of proteins, seeks to decode the expression and functionality of proteins, offering insights into the nuances of protein behavior and their critical role in health and disease. This discipline helps explain discrepancies between protein and mRNA levels, influenced by factors like post-translational modifications (Fig. 1). The establishment of the Human Proteome Organization (HUPO) and the Human Proteome Project (HPP) reflects the complexity of the proteome and underscores the necessity of global collaboration in advancing precision medicine [7,8]. HPP has pursued two goals: to credibly identify at least one isoform of every protein-coding gene; and to make proteomics an integral part of multiomics studies of human health and disease [9]. In 2005, the human liver proteome project proposed a comprehensive strategy, which included expression profile, modification profile, proteome localization map, protein–protein interaction (PPI) map, panel of antibodies, specimen bank and database warehouse, as well as physiome and pathome studies [10]. By capturing the spatiotemporal dynamics of protein expression, post-translational modifications and PPIs, proteomics provides a powerful foundation for biomarker discovery, clinical decision-making, and the implementation of personalized medicine (Fig. 2).

Over the past 15 years, global proteomics research has significantly expanded, evidenced by the rise in publications from 2009 to 2023 (Fig. S2A). This surge highlights the growing importance of proteomics in the scientific realm. While genomics has traditionally led in precision medicine, proteomics is rapidly gaining prominence, particularly since 2019, as it becomes more associated with precision medicine research. Proteomics interlinks with key pillars such as protein expression profiling, post-translational modifications, PPIs and spatial proteomics, among others (Fig. S2B). This progression not only marks a new era where proteomics is becoming a central force in precision medicine, but also lays the foundation for an evolved paradigm known as ‘phronesis medicine’—a wisdom-oriented clinical framework that emphasizes holistic integration of molecular data with contextual factors. This emerging approach underscores the potential of proteomics to transform healthcare beyond traditional precision paradigms through more nuanced clinical decision-making.

## KEY TECHNOLOGIES IN PROTEOMIC MEDICINE

Sydney Brenner, a distinguished Nobel laureate in physiology and medicine, proposed the ‘comprehensive, accurate and permanent’ (CAP criteria) as the gold standard for big scientific programs [11]. In the context of proteomics research, meeting these criteria involves a thorough analysis and detailed understanding of protein sequences and their functions, along with precise spatial and temporal quantification. Furthermore, the data, insights and principles derived from proteomics must maintain their validity over extended periods, ensuring their ongoing relevance and value. Clinical proteomics faces additional challenges such as: managing diverse sample types like frozen tissues, formaldehyde-fixed paraffin-embedded sections and body fluids, which complicate sampling strategies; demanding of extreme sensitivity by the wide dynamic range in body fluids; the need for high throughput in multicenter studies and large cohort proteomes; and the requirement for precise, powerful data-mining technology to handle big data. Despite these hurdles, significant advancements have been made in the field.

### Protein expression profile

In the last three decades, proteomic identification and quantification have greatly advanced due to new technologies, reagents, software and instruments. These include improvement in sample preparation, peptide separation, mass spectrometry (MS) sequencing, data analysis and the use of big data and deep learning. Innovations like pressure cycle technology (PCT) [12] and single-pot solid-phase-enhanced sample preparation (SP3) [13] improved the throughput and automation of sampling at the microscale. Multidimensional fractionation techniques [14] have improved proteome coverage by replacing traditional electrophoresis. Flexible and efficient MS-based techniques have been developed to meet different application scenarios of medical proteomics, mainly protein identification by pinpointing of cleaved peptide fragments (the bottom-up approach) [15] and the direct measure of intact protein and proteoforms (the top-down approach) [16]. Technologies like iTRAQ and TMT have improved the throughput and quantitative stability [17], despite their costs and potential distortions. The development of label-free quantification and data-independent acquisition (DIA), along with high-speed, precise MS, has further boosted throughput, coverage and reproducibility of proteomic analysis [18]. By integrating cutting-edge MS technology with single-cell sample processing, it is now possible to quantify over around 5000 proteins from a single HeLa cell with high throughput [19,20]. These technological breakthroughs in proteomics have enabled the creation of comprehensive reference maps of proteomes across a wide range of model organisms, providing valuable insights into protein expression and function [21–23].

### Post-translational modification profile

Proteins undergo post-translational modifications (PTMs), adding chemical groups to amino acid side chains, significantly diversifying their functions beyond their primary sequences. Key PTMs include phosphorylation, acetylation, ubiquitination, glycosylation and lactylation. The PTM code—comprising writers (adding PTMs), erasers (removing PTMs) and readers (interpreting PTMs)—regulates protein functions and cellular responses. PTMomics, the study of the roles of PTMs in cell regulation, highlights their potential in understanding complex biological processes and developing individualized treatments.

Discovered in 1883, phosphorylation is pivotal in precision medicine. The concept of reversible protein phosphorylation, introduced by Fischer and Krebs in 1964, earned them the Nobel Prize in 1992. Advancements in MS have propelled PTM analysis, identifying thousands of phosphorylation sites [24,25], which has revolutionized cancer treatment via kinase inhibitors. To date, the Food and Drug Administration (FDA) has approved 72 unique small-molecule kinase inhibitors for clinical use [26], with ongoing advancements promising further personalized cancer therapies.

Acetylation, one of the most important acylations, was discovered in 1963 on histones, later expanded to include other proteins like HMG and tubulin. Significant developments in the 1990s included the discovery of acetylation on p53 and identification of histone acetyltransferases (HATs) and histone deacetylases (HDACs). The bromodomain was recognized as an acetyl-lysine reader domain, leading to potent deacetylase inhibitors [27]. Acetylation proteomics has evolved to encompass the identification and quantification of acetylated proteins and their specific sites through sophisticated analytical techniques, including MS, enhanced by antibody-based enrichment strategies [28]. Researchers have since delved into the functional implications of acetylation across a spectrum of biological processes and disease states, underscoring its vital role in modulating cancer cell growth, metabolism and drug resistance [27,29,30]. This exploration has significantly contributed to our understanding of acetylation's multifaceted roles in cellular regulation and disease pathogenesis.

Ubiquitination, critical for protein degradation, cellular functioning and signal transduction, has become a key player in disease pathology, positioning it as a compelling target for therapeutic intervention. Innovative therapeutic strategies, including proteasome inhibitors, modulation of E3 ligases and deubiquitinating enzyme (DUB) inhibitors, show promising prospects. The field of ubiquitinomics has rapidly advanced with artificial ubiquitin-binding domains (UBDs) [31,32] and site-specific antibodies [33], enabling precise studies of ubiquitination. Innovations like UbiFast [34] and diGly antibody-based enrichment [35] have significantly improved ubiquitination analysis, identifying thousands of ubiquitination sites. Emerging strategies and technologies, such as proteolysis targeting chimeras (PROTACs) [36], chemical biology approaches and high-throughput screening, are opening new frontiers in the search for novel therapeutics.

Glycosylation, the most widespread and intricate PTM in mammals, has historically posed challenges due to glycan complexity. The glycosylation of many proteins, such as PD-L1, can significantly impact the effectiveness of antibody detection and therapeutic outcomes [37]. Advances in -omics and glycopeptide identification techniques now allow for detailed analysis of glycosylation sites and glycan compositions (ideally chemical structures), enhancing our understanding of their functional roles [38–40]. Spatial glycoproteomic analysis reveals the impact of AXL extracellular domain shedding on pancreatic cancer metastasis and proposes a combined inhibition strategy [41]. The strategic application of glycosylated ligands in drug delivery systems has advanced therapeutic precision, as demonstrated by the clinical development of hepatocyte-targeted GalNAc-siRNA conjugates, which utilize triantennary *N*-acetylgalactosamine to enable receptor-mediated RNAi therapy for liver-associated diseases [42].

Protein lactylation has recently emerged as an intriguing and significant PTM, characterized by the covalent attachment of lactate groups to lysine residues. This modification can have profound effects on protein structure, stability, function and interactions with other biomolecules [43]. As a relatively novel area of study, lactylation proteomics focuses on identifying lactylated proteins within biological samples, mapping specific lactylation sites and elucidating the functional consequences of these modifications [44]. Notably, aberrant lactylation is frequently linked to various pathological conditions, suggesting that dysregulation of lactate-derived lactylation plays a critical role in disease progression [45–47]. Given this, targeting lactate metabolism and its associated PTMs has gained significant attention as a promising therapeutic strategy, particularly in cancer and metabolic diseases. This expanding field holds great potential for the development of novel treatments by modulating protein lactylation and lactate dynamics.

The exploration of PTM code and PTMomics offers tremendous promise for deepening our understanding of disease biology and enhancing pharmaceutical development. With over 650 types of protein modifications identified [48], ongoing technologies and computational advancements will expand the inventory of novel PTMs. The insights gained from studying PTM-mediated disease mechanisms and identifying unique PTM signatures pave the way for precision medicine and the creation of targeted drugs [49]. Moreover, the continuous advancement in PTMomics techniques, coupled with their integration with other -omics disciplines, is poised to significantly propel the field forward.

### PPI network (protein linkage map)

Emergence occurs when a complex entity has properties or behaviors that its parts do not have on their own, and emerge only when they interact in a wider whole [50]. The PPIs form the foundation of protein complexes and machinery, establish hierarchical and cluster organization of protein community and give rise to emergent properties that a single protein doesn't bear. Notably, these emergent properties of PPIs occur on various levels of hierarchy, ranging from molecular to cellular, tissue/organ and organism scales. The emergence of characteristic life properties at each level derives from the emergent properties of the preceding level. Globally delineating protein interaction networks at different levels is a major task of proteomics, enabling resolution of the emergent phenotypes or features that manifest solely through protein population. The whole human interactome covers approximately 19 411 protein-coding genes [9], with an estimated scope of range 500 000 to 3 000 000 binary protein interactions [51].

A few technologies have been developed to systematically investigate protein complexes or binary PPIs, including yeast two-hybrid [52], affinity purification coupled with MS [53], and chemical cross-linking [54] etc. PPI networks have provided insights into the emergent properties of human protein society across various levels. At the intracellular level, the social architecture and module structure of the protein interaction network has been identified, such as DNA replication, RNA transcription, protein translation, protein transportation etc., implying the existence of highly connected topological modules or clusters in the network. Global landscapes or reference maps of the human interactome are an essential foundation to both molecular and systematic biology [52–54]. Furthermore, the intercellular network has been systematically investigated at the intracellular level for human immune cells, offering a systematic view of the intercellular wiring of these cells [55,56]. Moreover, the tissue- or organ-specific functional properties are largely attributed to the distinct interactome network of alternative splicing of proteins [57] or tissue-specific expressed proteins [52,58].

The abnormal emergent properties of protein society lead to human diseases. Re-wiring or abnormal assembling of protein complexes or protein machines give rise to unwanted emergent features, ultimately leading to dysfunctional phenotypes in various human diseases [59]. By comparing mutated and normal protein assemblies, new candidate disease interactions can be inferred, such as in the case of cancer [59,60] and autism [61]. Additionally, the pathogens cause abnormal emergence of protein society and lead to human diseases. Numerous studies have systematically investigated interaction networks between pathogens and human proteins, including SARS-CoV-2 [62] and Ebola virus [63] etc. These studies provide valuable resources, potential drug targets and candidate drugs for the clinical treatment of infectious diseases.

Protein–protein interactomics has emerged as a key enabling technology in precision medicine, providing systems-level understanding of disease mechanisms and facilitating the development of personalized therapeutic strategies. In oncology, interactome analyses have elucidated how cancer cells hijack normal protein interaction networks to establish oncogenic PPIs (oncoPPIs)—drivers of tumorigenesis and progression [60]. These oncoPPIs, frequently caused by somatic mutations, are now established molecular hallmarks of cancer. Advanced MS-based interactomics has enabled high-resolution mapping of these dysregulated interfaces, revealing novel druggable targets and informing the design of PPI-specific inhibitors [64–66].

In drug repurposing, network medicine exploits the human interactome to predict new therapeutic applications for existing drugs by evaluating the network proximity between disease-associated genes and drug targets. A prominent example is the COVID-19 pandemic, where interactome-based studies rapidly pinpointed host proteins interacting with SARS-CoV-2, uncovering several FDA-approved drugs with repurposing potential [67]. Beyond viral infections, modular network analyses have shown that expanding drug targets through their PPI neighbors broadens disease coverage, while proximity-based approaches have proved to be effective in prioritizing candidates with optimal efficacy and safety profiles for complex diseases like cardiovascular disorders [68].

For biomarker discovery, interactomics focuses on disease-associated hub proteins and dynamically altered subnetwork modules whose connectivity changes reflect disease states. In clinical settings such as cancer, PPI-guided analyses have yielded core regulatory proteins with strong prognostic or diagnostic value, enhancing specificity while streamlining candidate selection [69].

Notably, integrating patient-specific -omics data with global interactome frameworks has enabled the construction of personalized interactomes, capturing unique molecular disease signatures. For instance, individualized PPI networks from hypertrophic cardiomyopathy (HCM) patients successfully discriminated disease subtypes and identified fibrosis-related expression patterns, demonstrating the feasibility of network-based stratification [70]. Similar approaches are being explored in oncology, highlighting the transformative potential of PPI-driven analyses for tailored precision medicine interventions.

### Spatial proteome (proteome localization map)

Given the intrinsic link between a protein's precise location—whether at the tissue, cellular or subcellular level—and its function, it is imperative to examine protein expression with detailed spatial resolution. Spatial proteomics research offers a comprehensive insight into the distribution, interaction and functionalities of proteins. This approach holds considerable promise for advancing various medical fields.

Spatial proteome biology, essential for high-precision sampling and data analysis, is pioneering clinical insights by linking proteome features with specific locations. Discoveries like MYH10 and MYH11 biomarkers for atherosclerosis were made using 3D imaging of solvent-cleared organs profiled by MS (DISCO-MS), highlighting regional differences in plaque microenvironment [71]. Due to the well-known existence of molecular heterogeneity within tumor microenvironments and the accumulating inconsistency between mRNA and protein levels, laser capture microdissection (LCM)-based micrometer-resolved sampling coupled with differential proteomics is considered an efficient way to compare molecular features among tumor compartments, such as stromal NNMT's role in ovarian cancer [72] and the discovery of protein-enriched regions and chemo-resistance patterns on glioblastoma [73]. The above results suggest the value of region-specific proteomics in identifying disease drivers for targeted therapy.

Cell-type and single-cell spatial proteomics pose greater challenges in sensitivity and resolution compared to regional analysis. Techniques like fluorescence-activated cell sorting (FACS) isolating distinct cells from various histology layers of skin and for spatially and cell type-resolved proteomic profiling have revealed a distribution gradient of structural and immune-related proteins across skin layers and cellular subsets, offering insights into more precise pathology and treatment [74]. Additionally, a method utilizing cKit gradients in liver vessels sorted endothelial cells, uncovering an unexpected zonation of receptor tyrosine kinase Tie1 as a key regulator in sustaining efficient liver regeneration [75]. Advancements like deep visual proteomics (DVP) identified proteomic changes in various cell types from normal cells to invasive melanoma and highlighted key dysregulated pathways in tumor progression. DVP's application to extracellular matrix dynamics further showcases its potential beyond single-cell transcriptomics [76]. Not restricted to tumor-related disease, DVP has also revealed pronounced interferon signatures and STAT1 phosphorylation in both immune cells and keratinocytes of toxic epidermal necrolysis patients [77], pinpointing therapeutic targets for this lethal drug-induced skin disease. Even higher resolution can be achieved by single-cell DVP (scDVP), which is capable of single-cell proteome analysis without prior knowledge of the cell types [78]. scDVP has demonstrated cell-autonomous α1-antitrypsin accumulation with minimal intercellular stress propagation in α1-antitrypsin deficiency (AATD)-associated liver fibrosis, and highlighted peroxisomal upregulation as an early intervention target by PPAR-α agonist treatment for this disease [79]. These technological leaps generate unprecedented multidimensional datasets that ultimately demand a new clinical decision-making paradigm, phronesis medicine, which synthesizes high-resolution molecular profiles with clinical wisdom.

### Proteomics resources

As the proteomics landscape continues to evolve, the paradigm from data to information to knowledge to wisdom is exemplified by the collection and utilization of vast datasets from repositories like PRIDE [80], iProX [81] and MassIVE-KB [82], which transform into qualitative and quantitative information through tools like pFind [83], MaxQuant [84] and DIA-NN [85]. This information deepens into knowledge about biological processes and disease mechanisms via resources like dbPTM [48], BioGRID [86] and The Human Protein Atlas [87]. Ultimately, this knowledge guides clinical wisdom, enabling tailored treatments that enhance patient outcomes. Emphasizing collaboration across disciplines, this ‘data–information–knowledge–wisdom’ pipeline underscores the transformative impact of integrated proteomic resources in achieving personalized healthcare (Table 1).

**Table 1.** A summary of the main proteomics resources for proteomics-driven precision medicine.

Resource	Category	Features	Link
PRIDE [80]	MS data repository	Public and private MS raw data with metadata	https://www.ebi.ac.uk/pride/
iProX [81]	MS data repository	Public and private MS raw data with metadata	https://www.iprox.org/
MassIVE-KB [82]	MS data and peptide repository	Public and private MS raw data and peptide identifications with metadata	https://massive.ucsd.edu
PeptideAtlas [88]	Peptide repository	Curated peptide identifications and protein sequence assignments, high-quality and uniform reprocessing pipeline	https://peptideatlas.org/
UniProt [89]	Protein sequence repository	High-quality, comprehensive and freely accessible resource of protein sequence and functional information	https://www.uniprot.org/
SEQUEST [90]	MS data analysis tool	The first identification tool for proteomics data analysis	http://fields.scripps.edu/yates/wp/?page_id=17
MSFragger [91]	MS data analysis tool	A widely-used open search tool for proteomics data identification	https://msfragger.nesvilab.org/
pFind [83]	MS data analysis tool	A widely-used open search tool for proteomics data identification	http://pfind.org/index.html
MaxQuant [84]	MS data analysis tool	One of the most popular workflows for proteomics data identification and quantification	https://www.maxquant.org/
DIA-NN [85]	MS data analysis tool	A widely used tool for DIA proteomics data identification and quantification	https://github.com/vdemichev/DiaNN
Firmiana [92]	MS data analysis tool	A one-stop proteomic cloud platform for proteomics data processing and analysis	http://firmiana.org/ https://phenomics.fudan.edu.cn/firmiana
PANDA [93]	MS data analysis tool	A flexible and comprehensive tool for proteomics data quantification	https://sourceforge.net/projects/panda-tools/files/
dbPTM [48]	PTM repository	An integrated resource for protein PTMs, providing disease association based on nsSNPs	https://awi.cuhk.edu.cn/dbPTM/
UbiBrowser [94]	PTM knowledge base	A comprehensive platform for proteome-wide known and predicted ubiquitin ligase/deubiquitinase–substrate interactions in eukaryotic species	http://ubibrowser.ncpsb.org.cn
ExpressVis [95]	Protein expression analysis tool	A biologist-oriented interactive web server for exploring -omics data	https://omicsmining.ncpsb.org.cn/ExpressVis
BioGRID [86]	PPI repository	A biological general repository for PPI: archiving and disseminating genetic and protein interaction data from model organisms and humans	https://thebiogrid.org/
STRING [96]	PPI repository	A comprehensive database of known and predicted PPIs	https://string-db.org/
Cytoscape	PPI network analysis tool	One of the most popular network analysis tools	https://cytoscape.org/
BATMAN-TCM	TCM compound–protein interaction analysis tool	A comprehensive platform for the molecular mechanism research of TCM	http://bionet.ncpsb.org.cn/batman-tcm/
DCABM-TCM	TCM blood ingredient-protein interaction analysis tool	A comprehensive resource for TCM ingredients experimentally detected in the blood	http://bionet.ncpsb.org.cn/dcabm-tcm/
SPDB	Single-cell proteomic database	The current version contains 133 antibody-based single-cell proteomic datasets involving more than 300 million cells and over 800 marker/surface proteins, and 10 MS-based single-cell proteomic datasets involving more than 4000 cells and over 7000 proteins	https://scproteomicsdb.com/
ProteomicsDB [97]	Protein knowledge base	A comprehensive and multiorganism resource containing proteomics data and analysis tools	https://www.proteomicsdb.org/
Proteome of Life [23]	Protein knowledge base	The proteome landscape of 100 taxonomically diverse organisms with two million peptide and 340 000 stringent protein identifications obtained in a standardized manner	https://proteomesoflife.org/
HPP Portal [9]	Human proteome knowledge base	The official website of the Human Proteome Project since 2025	https://hppportal.net/index.html
The Human Protein Atlas [87]	Protein knowledge base	Tissue-specific and disease-specific protein expression data with immunohistochemistry images, high-quality protein annotations	https://www.proteinatlas.org/
Liverbase [98]	Human liver protein knowledge base	Proteomic datasets of heathy human liver	http://liverbase.hupo.org.cn/
IEDB [99]	The Immune Epitope Database	Catalogs experimental data on antibody and T cell epitopes studied in humans and other animal species in the context of infectious disease, allergy, autoimmunity and transplantation	https://www.iedb.org/
CPTAC Data Portal [100]	MS data repository for cancer proteomics	Large-scale proteomic datasets from various cancer types	https://proteomics.cancer.gov/data-portal
TTD	Therapeutic target database	An integrated resource for drug targets	https://idrblab.net/ttd/
Landscape of therapeutic targets	Therapeutic target database	A web server for therapeutic targets based on CPTAC pan-cancer data	https://targets.linkedomics.org/
POPPIT	Prediction tool for therapeutic targets	A web server for human genome-wide protein druggability prediction specially for protein and peptide drugs	http://poppit.ncpsb.org.cn/

nsSNP, non-synonymous single nucleotide polymorphism; TCM, traditional Chinese medicine.

## REFERENCE MAP OF THE HUMAN PROTEOME

Progress in the HPP has significantly advanced our understanding of the human proteome [21,22,101,102]. As of recent reports, 18,138 out of the 19 411 GENCODE protein-coding genes (93%) have been experimentally validated at the protein level, primarily through MS-based approaches [9]. The chemical and functional diversity of proteins is manifested through their many proteoforms, which are specific molecular forms of a protein product derived from a single gene. These proteoforms encompass all combinatorial variations arising from genetic polymorphisms, alternative RNA splicing, and PTMs [103,104].

Organs comprise multiple cell types organized in a complex hierarchy. Extensive research is now providing a spatial framework that maps the localization of proteins within the human body and animal models, from entire organs to the cells and organelle level. This includes detailed studies of protein expression [105], phosphorylation [106], proteoform [107] and PPIs [75], among others (Fig. S3). To better understand how trillions of cells and thousands of cell types contribute to a symphony of physiology and the mechanisms underlying disease, several proteomic studies focusing on specific anatomical regions or cell types using MS-based techniques were conducted. They offer vital insights into the cellular and molecular landscapes and the interactive networks within vital organs such as the heart [108], brain [109], skin [74] and liver [110]. Such approaches illuminate the fundamental principles of cellular cooperation within organs. For example, a cell-type-resolved proteomic study of the liver disclosed a division of labor between parenchymal cells and non-parenchymal cells, wherein the former predominantly manage pathway components while the latter initiate these pathways. Importantly, ongoing interactions between these cell types are essential for maintaining the functional identity of the parenchymal cells [110]. Additionally, a recent systematic analysis has revealed the diversity of human synaptic proteomes and their organization across brain regions and cell types, showcasing ∼1800 unique synapse-enriched proteins, common synaptic modules and specialized proteomic hotspots across 18 distinct synapse types [111]. A groundbreaking study has established the comprehensive human proteome distribution atlas spanning 21 tissues and 8 blood cell types. This resource enables precise mapping of plasma proteins to their tissue origins, unveils the disease-associated protein dynamics, and offers a novel perspective for elucidating the tissue origins of the plasma proteome and the dynamic changes associated with disease [112].

## HEALTH MONITORING AND DISEASE SURVEILLANCE BY PROTEOME

Health transcends the simple absence of illness, embodying a dynamic state of equilibrium across the body's systems. The blood proteome, easily accessible through non-invasive collection methods, offers significant potential for individualized health tracking. Traditional methods like chemiluminescence and enzyme-linked immunosorbent assay (ELISA) are inadequate for simultaneous detection of multiple biomarkers linked to specific diseases. To meet this demand, several advanced proteomics technologies have emerged, including proximity extension assay (PEA) [113], slow off-rate modified aptamer assay (SOMAmer), bead-based arrays and electrochemiluminescence (ECL) multiarray. Each of these technologies presents unique strengths in terms of proteome coverage, detection limits, sample throughput and assay duration (Table 2). Consequently, choosing the right technology is vital for the discovery, validation and clinical application of biomarkers. For instance, technologies like PEA, SOMAmer and MS offer extensive proteome coverage and high sensitivity, making them ideal for identifying potential biomarkers. In contrast, technologies suited for clinical testing, such as bead-based assays, single-molecule arrays and ECL should prioritize sensitivity, speed, reproducibility and cost-effectiveness.

**Table 2.** Performance and applications of proteomic technologies for multiplexed analysis of plasma proteins.

Technique	Principle	Clinical application	Plasma protein number/assay	Limit of detection	Dynamic range
ELISA [114]	Immunoassay	FDA approved	1	pg/mL–ng/mL	3–5 logs
CL [115]	Immunoassay	FDA approved	1	fg/mL	4–6 logs
ECL [116]	Immunoassay	FDA approved	1–10	fg/mL	4–6 logs
Simoa [117]	Immunoassay	FDA approved	1–10	fg/mL	>4 logs
NULISA [118]	Immunoassay	LDT	1–250	fg/mL	12 logs
Bead-based array [119]	Immunoassay	LDT	1–500	pg/mL	3–5 logs
MS [120]	MS-DIA-based	LDT	300–800	ng/mL	4–6 logs
PEA [113]	Immunoassay	LDT	1–5400+	fg/mL	10 logs
SOMAmer [121]	Aptamers-based	LDT	1–11 000	pg/mL	10 logs
Planar microarray [122]	Immunoassay	LDT	21 000+ (autoantibody)	pg/mL–ng/mL	3–5 logs

CL, chemiluminescence; LDT, laboratory-developed test; Simoa, single molecule array; NULISA, nucleic acid linked immuno-sandwich assay.

Innovative techniques, such as micro-sampling and wearable devices for collecting hourly blood samples over a week, have unveiled notable molecular variability [123]. These variations, tied to physiological changes and physical exertion, underscore the ability of proteomics to monitor health on a short-term basis by tracking hourly shifts in the body's molecular and physiological states. Delving into plasma proteins across the human lifespan, proteomics provides a detailed view of the ebbs and flows from birth through to old age. Early studies have uncovered distinct, rhythmic changes in protein abundance that align with aging and the onset of diseases. The significant work has led to the development of 11 aging models that can forecast health and disease states for nine age-related conditions, including Alzheimer's disease, atrial fibrillation, cerebrovascular disease, diabetes, heart attack, hypercholesterolemia, hypertension, obesity and gait disorders [124]. The UK Biobank Plasma Proteomics Project (UKB-PPP) has further advanced this field by conducting large-scale plasma proteomics research within a uniquely comprehensive cohort of over 500 000 individuals. Leveraging data from the UKB-PPP, researchers explored the associations of plasma proteomics with health and developed a proteomic aging clock that predicts mortality and the risk of common age-related diseases across diverse populations [125,126]. Moreover, proteomic prediction models outperformed clinical models in assessing the 10-year risk of 67 diseases across various pathologies, such as multiple myeloma, motor neuron disease, pulmonary fibrosis, celiac disease and dilated cardiomyopathy [127]. These advances are underpinned by a systematic proteome-phenome atlas that maps 2920 plasma proteins to 1066 diseases/traits, revealing over 650 pan-disease proteins, 1000+ sex/age-heterogeneous proteins, and 183 diseases with AUC > 0.80 protein-based diagnostics, while identifying 474 causal proteins for drug repurposing via protein quantitative trait loci (pQTL) integration [128].

In a notable cohort study, researchers devised the protein risk scores that outperformed traditional polygenic risk scores in predicting atherosclerotic cardiovascular disease [129] and diabetes [130], respectively. Another study established a nine-protein risk score that surpassed existing cardiovascular risk scores in predicting heart-related events [131]. Additionally, by analyzing plasma proteomic data, levels of glial fibrillary acidic protein (GFAP) were identified as predictors of dementia up to 15 years before onset, showcasing the profound potential of proteomics in pre-emptive disease risk assessment [132]. Significantly, regulatory bodies, such as the FDA, have approved multiple protein biomarkers for cancer detection, including OVA1 for ovarian cancer risk evaluation [133], DCP and AFP-L3 for hepatocellular carcinoma diagnosis [134] and CancerSEEK for eight cancer types of detection [135] etc., leveraging proteomics to enhance existing markers (Table S1).

## CUSTOMIZED TREATMENT BASED ON THE PROTEOME

Over the past decade, the clinical application of protein markers, such as PD-L1, HER2 and CLDN18.2 to guide treatment decisions has led the medical community to increasingly acknowledge the significant heterogeneity among patients diagnosed with the same condition. This has highlighted the fact that disease manifestations and responses to treatment are uniquely individualized, emphasizing the need for personalized therapeutic approaches. This realization has propelled proteomic classifications to the forefront as an essential instrument for unraveling this complexity and informing therapeutic choices, including but not limited to breast cancer [136,137], liver cancer [138], LIF for pancreatic adenocarcinoma [139], lung cancer [140], gastric cancer [141], esophageal cancer [142] and ampullary adenocarcinoma [143] (Table S2). Diverging from genomic or transcriptomic approaches, proteomic subtyping offers a nuanced perspective, particularly evident in the prognosis of various cancers such as liver [138] and lung adenocarcinoma [140]. It highlights subtype-specific pathways and proteins as pivotal targets for drug development, such as SOAT1 for hepatocellular carcinoma [138], NNMT for ovarian cancer [72] and DPYD and TYMP for gliomas [144], respectively, thereby offering a more tailored strategy in patient care. To expand access to breakthrough cancer treatments using checkpoint inhibitors for a broader range of patients, immunopeptidomics-driven tumor antigen peptide discovery has significantly advanced CAR-T immunotherapy and tumor vaccines. These innovative approaches are playing an increasingly important role in the management of malignancies such as neuroblastoma [145], glioblastoma [146] and other cancers, offering promising new avenues for treatment.

Drug resistance poses a significant challenge in treating various diseases, highlighting the need for innovative therapies to overcome resistance and enhance treatment efficacy. The proteogenomic study of 242 high-grade serous ovarian cancers showed high levels of fatty acid oxidation proteins in chemotherapy-resistant cases. Targeting CPT1A, a key enzyme in this pathway, enhanced sensitivity to platinum-based chemotherapies [147]. Similarly, a proteomic analysis discovered that proteostasis reprogramming is central to KRAS inhibitor (KRASi) resistance. Reactivating IRE1α, crucial for maintaining proteostasis, offers a promising target to counteract KRASi resistance [148]. This growing recognition of therapy-specific resistance mechanisms further validates the phronesis medicine model, which requires continuous adaptation of treatment strategies based on real-time proteomic monitoring and clinical context reevaluation.

## THE CHALLENGE AND PROMISE OF PROTEOMICS-DRIVEN PHRONESIS MEDICINE

Despite advances in precision medicine, optimally treating each patient is complicated by the complexity of medical specialties, technological growth and diverse diseases and guidelines. Challenges such as diagnostic uncertainty, the gap between knowledge and practice, and cognitive biases among clinicians complicate achieving consensus on best practices, highlighting the need for virtues like Aristotle's concept of Phronesis to manage uncertainty and apply knowledge effectively [149]. Here we propose phronesis medicine as an evolutionary advancement of precision medicine, building upon its molecular foundation by incorporating clinical wisdom and holistic patient dynamics to achieve truly optimized care.

Cross-disciplinary approaches are revolutionizing clinical decision-making by adopting a holistic view of the complex human body, involving systematic analysis from cellular to organ levels and their interactions [150]. Utilizing artificial intelligence (AI), especially deep reinforcement learning, has shown superior decision-making capabilities in various fields and is proving effective in managing complex medical conditions, suggesting a significant role for AI in future healthcare advancements [151,152] (Fig. S4).

To advance phronesis medicine, we initiated the international Proteomic Navigator of the Human Body (π-HuB Project) with three main objectives [153]. First, to map the human proteome's ‘anatomy space’ using advanced techniques like single-cell and spatial proteomics, detailing tissue and cellular protein compositions and their interactions. Second, to define the proteome's ‘state space’ by tracking proteomic changes across an individual's life and under various conditions. Lastly, to develop a π-HuB navigation system that identifies body states, traces lineage through dynamic models, and plans optimal treatment sequences, positioning this as a foundational step towards proteomics-driven phronesis medicine.

## Supplementary Material

nwaf278_Supplemental_Files
