# Supplementary material for: The coming era of proteomics-driven precision medicine
Source: Natl Sci Rev. 2025 Jul 14;12(8):nwaf278. doi: 10.1093/nsr/nwaf278 (PMC12365760; doi:10.1093/nsr/nwaf278)
Supplement: nwaf278_Supplemental_Files [file nwaf278_supplemental_files.zip › supplementary figure legend.docx]

**Figure S1 | Estimated heritability for complex diseases in European populations in the UK Biobank**. The liability-scale heritability calculated by stratified linkage disequilibrium score regression (SLDSC) [1] that passed quality control were shown in 9 categories in descending order separately (https://pan.ukbb.broadinstitute.org/docs/heritability). Black error bars mark ±2 standard errors centered on the estimated heritability.

Reference

1. Finucane HK, Bulik-Sullivan B, Gusev A, et al. Partitioning heritability by functional annotation using genome-wide association summary statistics. *Nat. Genet****.*** 2015; **47**: 1228-1235.

**Figure S2 | Bibliometrics and research hotspots of proteomics, from 2009 to 2023.** a, The number of papers published in the field of proteomics in the past 15 years. b, The development and evolution of research hotspots in proteomics in the past 15 years. The development of the protein field in the past 15 years has been divided into three periods. According to the time, correlation and cited frequency, relevant documents are extracted, the data were de-duplicated, merged and cleaned, and the literature keywords were clustered by Vosviewer visualization software. We divided these words into words related to protein's omics technology and words related to diseases, From 2009 to 2013, there were related researches on diseases in proteomics. From 2014 to 2018, “precision medicine” became a hot spot in proteomics. From 2019 to 2023, the research on precision medicine in proteomics continued to deepen and expand.

**Figure S3 | Representative reference maps of human proteome across different resolution scales**. Circle color denotes species. The number of divisions in the circle represents the resolution level. Different colored fonts represent different dimensions of the proteome. MS, Mass spectrometry; TMT, Tandem mass tag; IMAC: immobilized metal ion affinity chromatography.

Reference

1. Chinese Human Liver Proteome Profiling Consortium. First insight into the human liver proteome from PROTEOME(SKY)-LIVER(Hu)1.0, a publicly available database. J. Proteome Res. 2010; 9:79-94.
2. Kim MS, Pinto SM, Getnet D, et al. A draft map of the human proteome. *Nature.* 2014; **509**:575-581.
3. Uhlén M, Fagerberg L, Hallström BM, et al. Proteomics. Tissue-based map of the human proteome. *Science.* 2015; **347**:1260419.
4. Doll S, Dreßen M, Geyer PE, et al. Region and cell-type resolved quantitative proteomic map of the human heart. *Nat Commun*. 2017; **8**:1469.
5. Sharma K, Schmitt S, Bergner CG, et al. Cell type- and brain region-resolved mouse brain proteome. *Nat Neurosci*. 2015; **18**:1819-1831.
6. Dyring-Andersen B, Løvendorf MB, Coscia F, et al. Spatially and cell-type resolved quantitative proteomic atlas of healthy human skin. *Nat Commun*. 2020; **11**:5587.
7. Ding C, Li Y, Guo F, et al. A Cell-type-resolved Liver Proteome. *Mol Cell Proteomics*. 2016; **15**:3190-3202.
8. van Oostrum M, Blok TM, Giandomenico SL, et al. The proteomic landscape of synaptic diversity across brain regions and cell types. *Cell*. 2023; **186**:5411-5427.e23.
9. Drown BS, Drown BS, Jooß K, et al. Mapping the Proteoform Landscape of Five Human Tissues. *J. Proteome Res.* 2022; **21**:1299-1310.
10. Su, P., McGee, J.P., Durbin, K.R., et al. (2022). Highly multiplexed, label-free proteoform imaging of tissues by individual ion mass spectrometry. Sci. Adv*.* *8*, eabp9929.
11. Melani RD, Gerbasi VR, Anderson LC, et al. The Blood Proteoform Atlas: A reference map of proteoforms in human hematopoietic cells. *Science*. 2022; **375**:411-418.
12. Giansanti P, Samaras P, Bian Y, et al. Mass spectrometry-based draft of the mouse proteome. *Nat Methods*. 2022; **19**:803-811.
13. Xiang H, Zhang B, Wang Y, et al. Region-resolved multi-omics of the mouse eye. *Cell Rep.* 2023; **42**:112121.
14. Inverso D, Shi J, Lee KH, et al. A spatial vascular transcriptomic, proteomic, and phosphoproteomic atlas unveils an angiocrine Tie-Wnt signaling axis in the liver. *Dev Cell*. 2021; **56**:1677-1693.e10.

**Figure S4 | The related areas of phronesis medicine.**

Reference

1. Sternberg, R. J. Wisdom: Its Nature, Origins, And Development. The Cambridge University Press, 1990.
2. Jeste DV, Lee EE. The Emerging Empirical Science of Wisdom: Definition, Measurement, Neurobiology, Longevity, and Interventions. *Harv Rev Psychiatry*. 2019; **27**:127-140.
3. MoTrPAC Study Group, Lead Analysts, And MoTrPAC Study Group. Temporal dynamics of the multi-omic response to endurance exercise training. *Nature*. 2024; **629**:174–183.
4. Vandamme D, Fitzmaurice W, Kholodenko B, et al. Systems medicine: helping us understand the complexity of disease. *QJM*. 2013; **106**, 891–895.
5. Kitano, H. Systems biology: a brief overview. *Science.* 2002; **295**, 1662–1664.
6. Bontemps-Hommen CMML, Baart A, and Vosman FTH. Practical wisdom in complex medical practices: a critical proposal. *Med. Heal. Care Philos*. 2019; **22**, 95-105.
7. Muench, J. Balint work and the creation of medical knowledge. *Int. J. Psychiatry Med*. 2018; **53**, 15-23.
8. Saraga M, Boudreau D, Fuks A. Engagement and practical wisdom in clinical practice: a phenomenological study. *Med. Heal. Care Philos*. 2019; **22**, 41-52.
9. McDonald TO, Cheng YC, Graser C, et al. Computational approaches to modelling and optimizing cancer treatment. *Nat. Rev. Bioeng*. 2023; **1**, 695-711.
10. Sutton, R. S. and Barto, A. G. Reinforcement learning: An introduction, 2nd ed. The MIT Press, 2018.
11. Mnih V, Kavukcuoglu K, Silver D, et al. Human-level control through deep reinforcement learning. *Nature.* 2015; **518**, 529–533.
12. Kaufmann E, Bauersfeld L, Loquercio A, et al*.* Champion-level drone racing using deep reinforcement learning. *Nature.* 2023; **620**, 982–987.
13. Komorowski M, Celi LA, Badawi O, Gordon AC, Faisal AA. The Artificial Intelligence Clinician learns optimal treatment strategies for sepsis in intensive care. *Nat Med*. 2018; **24**:1716-1720.
14. Wang G, Liu X, Ying Z, et al. Optimized glycemic control of type 2 diabetes with reinforcement learning: a proof-of-concept trial. *Nat Med*. 2023; **29**:2633-2642.
