# Supplementary material for: The coming era of proteomics-driven precision medicine
Source: Natl Sci Rev. 2025 Jul 14;12(8):nwaf278. doi: 10.1093/nsr/nwaf278 (PMC12365760; doi:10.1093/nsr/nwaf278)
Supplement: nwaf278_Supplemental_Files [file nwaf278_supplemental_files.zip › Supplementary_tables-250526.docx]

**Table S1. FDA or CE-approved and LDT protein biomarkers for cancer detection**

| **Test** | **Cancer** | **Clinical Utility** | **Clinical Index** | **Performance** | **Technology** | **Approval** | **Company** |
| --- | --- | --- | --- | --- | --- | --- | --- |
| **The 4Kscore® Score Test** [1] | Prostate cancer | For assessing aggressive prostate cancer risk in men with an abnormal PSA or DRE result | Total PSA, free PSA, intact PSA, and hK2 | AUC ranges from 0.80 to 0.90 | ECL | FDA (2021) | OPKO Health, Inc. |
| **CancerSEEK** [2] | Multiple types of cancer (breast, colorectal, gastric, liver, lung, esophageal, ovarian, or pancreatic cancer) | For early cancer detection | Sixteen genes and eight proteins (CA125, CEA, CA19-9, PRL, HGF, OPN, MPO, TIMP-1) | 69 to 98% sensitivity (depending on cancer type) and 99% specificity. | Gene: Illumina MiSeq or HiSeq 4000; Protein: bead-based arrays (Luminex) | FDA (2019) | Thrive Earlier Detection Corp. |
| **OVERA ®** [3] | Ovarian cancer | For assessing ovarian cancer risk in women diagnosed with ovarian tumor before a planned surgery | Five proteomic biomarkers (CA125, transferrin, ApoA-1, HE4, FSH) | 96% sensitivity (with clinical assessment) and 98% NPV | Chemiluminescence | FDA (2016) | Vermillion Inc. |
| **ROMA** [4] | Ovarian cancer | For classifying women with an adnexal mass as low- or high risk of being found with an ovarian malignancy at the surgery | CA125, HE4, and menopausal status | 75% specificity, 77% and 92% sensitivity in premenopausal and postmenopausal women, respectively | ELISA, Chemiluminescence | FDA (2011) | Fujirebio Diagnostics, Inc |
| **DCP and AFP-L3** [5] | Hepatocellular carcinoma | Aid in the risk assessment of patients with chronic liver disease for development of hepatocellular carcinoma | DCP and AFP-L3 | 75% sensitivity and over 94% specificity | Fluorescence immunoassay | FDA (2011) | Wako Diagnostics |
| **EarlyCDT®-Lung test** [6] | Lung cancer | For the early detection of lung cancer in high-risk patients | Seven autoantibodies (CAGE, GBU4-5, HuD, MAGEA4, NY-ESO-1, p53 and SOX2) | 90.4% specificity and 32.1% sensitivity for detecting lung cancer at 2 years | ELISA | CE | Freenome Holdings Inc |
| **Videssa® Breast** [7] | Breast cancer | For the detection of breast cancer | Six serum protein biomarkers (AFP, CA19-9, CEA, TNF-α, VEGF-C, ErbB2) and eleven tumor-associated Autoantibodies (ANXA1, ATF3, ATP6AP1, BAT4, BDNF, CTBP1, DBT, HOXD1, IGF2BP1, IGFBP2, ErbB2) | 93% sensitivity and 98% NPV in a population of women ages 25-75. | ELISA | LDT | Provista Diagnostics, Inc |
| **IMMray™ PanCan-d test** [8] | Pancreatic ductal adenocarcinoma | For the detection of early-stage pancreatic ductal adenocarcinoma | Nine serum protein biomarkers (CA125, IGFBP3, OPG, CST3, GSN, C5, CFB, C4, CA19-9) | 85% sensitivity/99% specificity (early stage I and II vs. healthy controls) | Planar microarray | LDT | Immunovia, Inc. |

Abbreviations: FDA: food and drug administration; CE: conformity of europe; LDT: laboratory developed test; ECL: electrochemiluminescence; ELISA: enzyme-linked immunosorbent assay; DRE: digital rectal exam; PSA: prostate-specific antigen; hK2: human kallikrein 2; CA125: cancer antigen 125; CEA: carcinoembryonic antigen; CA19-9: cancer antigen 19-9; PRL: prolactin; HGF: hepatocyte growth factor; OPN: osteopontin; MPO: myeloperoxidase; TIMP-1: tissue inhibitor of metalloproteinases 1; ApoA-1: apolipoprotein A-1; HE4: human epididymis protein 4; FSH: follicle-stimulating hormone; ROMA: risk of ovarian malignancy algorithm; DCP: des-gamma-carboxy prothrombin; AFP-L3: Lens culinaris agglutinin-reactive fraction of AFP; NPV: negative predictive value; AFP: Alpha-fetoprotein; TNF-α: Tumor necrosis factor; VEGF-C: Vascular endothelial growth factor C; ErbB2: Receptor tyrosine-protein kinase erbB-2, also known as human epidermal growth factor receptor 2 (HER2); ANXA1: Annexin A1; ATF3: Cyclic AMP-dependent transcription factor; ATP6AP1: V-type proton ATPase subunit S1; BAT4: G patch domain and ankyrin repeat-containing protein 1; BDNF: Brain-derived neurotrophic factor; CTBP1: C-terminal-binding protein 1; DBT: Lipoamide acyltransferase component of branched-chain alpha-keto acid dehydrogenase complex, mitochondrial; HOXD1: Homeobox protein Hox-D1; IGF2BP1: Insulin-like growth factor 2 mRNA-binding protein 1; IGFBP2: Insulin-like growth factor binding protein; IGFBP3: Insulin-like growth factor-binding protein 3; OPG: Tumor necrosis factor receptor superfamily member 11B; CST3: Cystatin C; GSN: Gelsolin; C5: Complement C5; CFB: Complement factor B; C4: Complement C4; CAGE: cancer associated antigen gene; GBU4-5:Atp-binding RNA helicase; HuD: Hu-antigen D; MAGEA4: melanoma antigen gene A4, NY-ESO-1: cancer/testis antigen 1; p53: Cellular tumor antigen p53; SOX2: Transcription factor SOX-2.

Reference

1. https://4kscore.com/science-behind-the-score/

2. https://www.science.org/doi/10.1126/science.aar3247

3. https://www.ajog.org/article/S0002-9378(16)00465-8/fulltext

4. https://www.ncbi.nlm.nih.gov/pmc/articles/PMC7239964/

5. https://www.ncbi.nlm.nih.gov/pmc/articles/PMC7246383/

6. https://www.ncbi.nlm.nih.gov/pmc/articles/PMC7806972/

7. https://aacrjournals.org/clincancerres/article/25/1/142/125168/A-Noninvasive-Blood-based-Combinatorial-Proteomic

8. https://www.ncbi.nlm.nih.gov/pmc/articles/PMC8963856/

**Table S2. Proteomics-driven disease subtyping and therapeutic targets**

| **Disease** | **Subtypes** | **Prognosis-related**  **signatures** | **Drug**  **response** | **Targets (Drug)** |
| --- | --- | --- | --- | --- |
| **LUAD/**  **LUSC/**  **SCLC** | Three subclasses [1]: Environment and metabolism high; Mixed type; Proliferation and proteasome  Three subclasses [2]: Acinar developmental; TDG & GPX2 high-level; immune-hot, inflammation & IFN signal  Five subclasses [3]: Basal-inclusive; EMT-enriched; Classical (KEAP1, CUL3, NFE2L2 genes mutation); Inflamed-secretory; Proliferative-primitive  Two subclasses [4]: Immune-enriched; Immune-deprived | HSP90AB1, IMPDH2, GAPDH [1]  MMP11, MMP7 [5]  PLA2G2A [6] |  | HSP90AB1 [1] |
| **ESCA** | Four subclasses: Cell cycle signaling activation; Oncogenic activation; Immune suppression; Immune modulation[7] | ELOA, SCAF4 [8]  CLK1 [9] | Immunotherapy response: ADD2, FGA, FGG, SPTB, ZC3H7B, LSR, NDUFB7, RNF214, WIPF2, NCS1 [10] | CLK1 (TG003) [11]  PGK1 S203 (Gemcitabine) [12] |
| **STAD** | Three subclasses [13]: Dysregulation in the cell cycle; Dysregulation in the cell cycle & EMT process; Worst survival & immune response proteins enriched & chemotherapy insensitive | THSD4 [13] | resistant to 5-FU, oxaliplatin, doce-taxel): THSD4 [13,14] |  |
| **COAD** | Three subclasses [15]: Increased RNA processing and DNA mismatch repair (MMR); ECM-receptor integration, focal adhesion proteins enriched & immune-related pathways proteins enriched; DNA replication and Metabolism | 9-protein model including PDP1, ALR, ENOG, NPC2, FYCO1, STXB1, ARH40, RIMC1, MTMR5 [16] | resistance to EGFR blockade: EPHA2 [17] | PLOD2(Minoxidil) [18]  RAF1 (Regorafenib) [15] |
| **HCC** | Three subclasses [19]: Metabolism; Proliferation; Immune & disrupted cholesterol homeostasis  Three subclasses [20]: Metabolism; Microenvironment; dysregulated; proliferation  Three subclasses [21]: Potential ICB beneficiaries; VEGFR2 high expressed & anti-angiogenesis therapy; Responds to mTOR inhibitors or MKI [21] | SOAT1 [19]  PYCR2, ADH1A [20] |  | SOAT1(Avasimibe) [19]  TOP1/TOP2A/TOP2B [20]  NUAK1/2, CHEK1 (WZ4003 and AZD7762) [22] |
| **PAAD** | Three subclasses [23]: Metabolic bioprocesses (glycolysis & TCA cycle); Blood coagulation; Cell cycle & worst prognosis |  | platinum resistance: UBE2T [24] | LIF [25,26] |
| **BRCA** | Five subclasses [27,28]: Luminal A; Luminal B; HER2-enriched; Basal-like; Normal breast-like |  |  | PI3K/mTOR(TAK-117/TAK-128) [29] TALDO1(AO-022) [30] |
| **OV** |  |  | platinum resistance: CPT1A [31,32]；  PARPi resistance: OXPHOS [33] | NNMT (5-amino-1-methylquinolin-1-ium) [34] |
| **KRAS mutant cancers** | Two subclasses [35]: Mesenchymal phenotype, stromal invasion, worse prognosis); Cyclin-dependent features and better survival outcomes |  |  | DOT1L, SHP2 (EPZ-5676 and EPZ004777) [36] |

Abbreviations: LUAD: Lung adenocarcinoma; LUSC: Lung squamous cell carcinoma; SCLC: Small cell lung cancer; ESCA: Esophageal carcinoma; STAD: Stomach adenocarcinoma; COAD: Colon adenocarcinoma; HCC: Hepatocellular carcinoma; PAAD: Pancreatic adenocarcinoma; BRCA: Breast invasive carcinoma; OV: Ovarian cancer.

Reference

1. Xu JY, Zhang C, Wang X, et al. Integrative Proteomic Characterization of Human Lung Adenocarcinoma. *Cell*. 2020; **182**:245-261.e17.

2. Soltis AR, Bateman NW, Liu J, et al. Proteogenomic analysis of lung adenocarcinoma reveals tumor heterogeneity, survival determinants, and therapeutically relevant pathways. *Cell Rep. Med.* 2022; **3**:100819.

3. Satpathy S, Krug K, Jean Beltran PM, et al. A proteogenomic portrait of lung squamous cell carcinoma. *Cell*. 2021; **184**:4348-4371.e40.

4. Yang L, Zhang Z, Dong J, et al. Multi-dimensional characterization of immunological profiles in small cell lung cancer uncovers clinically relevant immune subtypes with distinct prognoses and therapeutic vulnerabilities. *Pharmacol. Res.* 2023; **194**:106844.

5. Chen YJ, Roumeliotis TI, Chang YH, et al. Proteogenomics of Non-smoking Lung Cancer in East Asia Delineates Molecular Signatures of Pathogenesis and Progression. *Cell.* 2022; **182**: 226-244.e17.

6. Zhu H, Shi H, Lu J, et al. Proteomic profiling reveals the significance of lipid metabolism in small cell lung cancer recurrence and metastasis. *J Transl Med*. 2024; **22**:1117.

7. Liu Z, Zhao Y, Kong P, et al. Integrated multi-omics profiling yields a clinically relevant molecular classification for esophageal squamous cell carcinoma. *Cancer Cell*. 2023; **41**:181-195.e9.

8. Liu, W., Xie, L., He, Y.H., et al. Large-scale and high-resolution mass spectrometry-based proteomics profiling defines molecular subtypes of esophageal cancer for therapeutic targeting. *Nat. Commun*. 2021; **12**:4961.

9. Li Y, Yang B, Ma Y, et al. Phosphoproteomics reveals therapeutic targets of esophageal squamous cell carcinoma. *Signal Transduct. Target. Ther*. 2021; **6**: 381.

10. Ma F, Li Y, Xiang C, et al. Proteomic characterization of esophageal squamous cell carcinoma response to immunotherapy reveals potential therapeutic strategy and predictive biomarkers. *J Hematol Oncol*. 2024; **17**:11.

11. Schmidt N M, Wing PAC, Diniz MO, et al. Targeting human Acyl-CoA:cholesterol acyltransferase as a dual viral and T cell metabolic checkpoint. *Nat. Commun.* 2021; **12**: 2814.

12. Li L, Jiang D, Zhang Q, et al. Integrative proteogenomic characterization of early esophageal cancer. *Nat. Commun.* 2023; **14**:1666.

13. Ge S, Xia X, Ding C, et al. A proteomic landscape of diffuse-type gastric cancer. *Nat Commun*. 2018; **9**:1012.

14. Li Y, Xu C, Wang B, et al. Proteomic characterization of gastric cancer response to chemotherapy and targeted therapy reveals new therapeutic strategies. *Nat. Commun.* 2022; **13**:5723.

15. Li C, Sun YD, Yu GY, et al. Integrated Omics of Metastatic Colorectal Cancer. *Cancer Cell.* 2020; **38**:734-747.e9.

16. Xu K, Yin X, Chen H, et al. Prediction of overall survival in stage II and III colon cancer through machine learning of rapidly-acquired proteomics. *Cell Discov.* 2024; **10**:85.

17. Beekhof R, Bertotti A, Böttger F, et al. Phosphoproteomics of patient-derived xenografts identifies targets and markers associated with sensitivity and resistance to EGFR blockade in colorectal cancer. *Sci. Transl. Med.* 2023; **15**: eabm3687.

18. Shao Y, Xu K, Zheng X, et al. Proteomics profiling of colorectal cancer progression identifies PLOD2 as a potential therapeutic target. *Cancer Commun*. (Lond) 2022; **42**:164-169.

19. Jiang Y, Sun A, Zhao Y, et al. Proteomics identifies new therapeutic targets of early-stage hepatocellular carcinoma. *Nature*. 2019; **567**:257-261.

20. Gao Q, Zhu H, Dong L, et al. Integrated Proteogenomic Characterization of HBV-Related Hepatocellular Carcinoma. *Cell*. 2019; **179**:561-577.e22.

21. Fujita M, Chen MM, Siwak DR, et al. Proteo-genomic characterization of virus-associated liver cancers reveals potential subtypes and therapeutic targets. *Nat. Commun*. 2022;**13**:6481.

22. Golkowski M, Lau HT, Chan M, et al. Pharmacoproteomics Identifies Kinase Pathways that Drive the Epithelial-Mesenchymal Transition and Drug Resistance in Hepatocellular Carcinoma. *Cell systems.* 2020; **11**:196-207.e7.

23. Tong Y, Sun M, Chen L, et al. Proteogenomic insights into the biology and treatment of pancreatic ductal adenocarcinoma. *J. Hematol. Oncol*. 2022; **15**:168.

24. Jiang X, Ma Y, Wang T, et al. Targeting UBE2T Potentiates Gemcitabine Efficacy in Pancreatic Cancer by Regulating Pyrimidine Metabolism and Replication Stress. *Gastroenterology.* 2023; **164**:1232-1247.

25. Shi Y, Gao W, Lytle NK, et al. Targeting LIF-mediated paracrine interaction for pancreatic cancer therapy and monitoring. *Nature.* 2019; **569**:131-135.

26. Huang P, Gao W, Fu C, et al. Clinical functional proteomics of intercellular signalling in pancreatic cancer. *Nature*. 2025; **637**:726-735.

27. Perou C M, Sørlie T, Eisen MB, et al. Molecular portraits of human breast tumours. *Nature.* 2000; **406**:747-752.

28. Sorlie T, Perou CM, Tibshirani R, et al. Gene expression patterns of breast carcinomas distinguish tumor subclasses with clinical implications. *Proc. Natl. Acad. Sci. U. S. A.* 2001; **98**:10869-10874.

29. Huang KL, Li S, Mertins P, et al. Proteogenomic integration reveals therapeutic targets in breast cancer xenografts. *Nat. Commun*. 2017; **8**:14864.

30. Xu G, Huang R, Wumaier R, et al. Proteomic Profiling of Serum Extracellular Vesicles Identifies Diagnostic Signatures and Therapeutic Targets in Breast Cancer. *Cancer Res*. 2024; **84**:3267-3285.

31. Chowdhury S, Kennedy JJ, Ivey RG, et al. Proteogenomic analysis of chemo-refractory high-grade serous ovarian cancer. *Cell*. 2023; **186**:3476-3498.e35.

32. Huang D, Chowdhury S, Wang H, et al. Multiomic analysis identifies CPT1A as a potential therapeutic target in platinum-refractory, high-grade serous ovarian cancer. *Cell Rep. Med*. 2021; **2**:100471.

33. Burdett NL, Willis MO, Alsop K, et al. Multiomic analysis of homologous recombination-deficient end-stage high-grade serous ovarian cancer. *Nat. Genet*. 2023; **55**:437-450.

34. Eckert MA, Coscia F, Chryplewicz A, et al. Proteomics reveals NNMT as a master metabolic regulator of cancer-associated fibroblasts. *Nature*. 2019; **569**:723-728.

35. Chong W, Zhu X, Ren H, et al. Integrated multi-omics characterization of KRAS mutant colorectal cancer. *Theranostics*. 2022; **12**:5138-5154.

36. Liu Z, Liu Y, Qian L, et al. A proteomic and phosphoproteomic landscape of KRAS mutant cancers identifies combination therapies. *Mol. Cell*. 2021; **81**:4076-4090.e8.
