# Supplementary figures and images for: The coming era of proteomics-driven precision medicine
Source: Natl Sci Rev. 2025 Jul 14;12(8):nwaf278. doi: 10.1093/nsr/nwaf278 (PMC12365760; doi:10.1093/nsr/nwaf278)

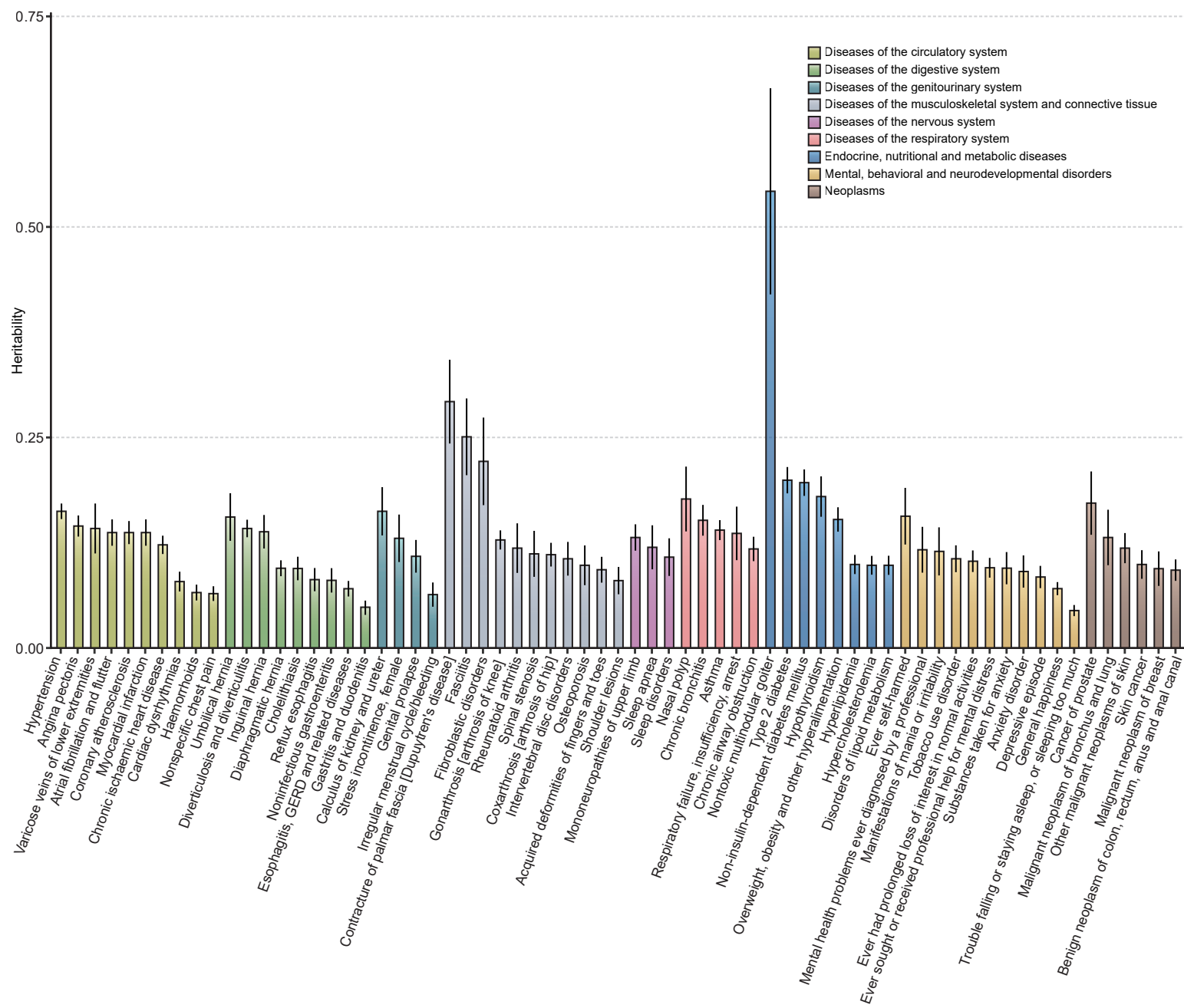

Supplement: nwaf278_Supplemental_Files [file nwaf278_supplemental_files.zip › Fig._S1.pdf]

**a**

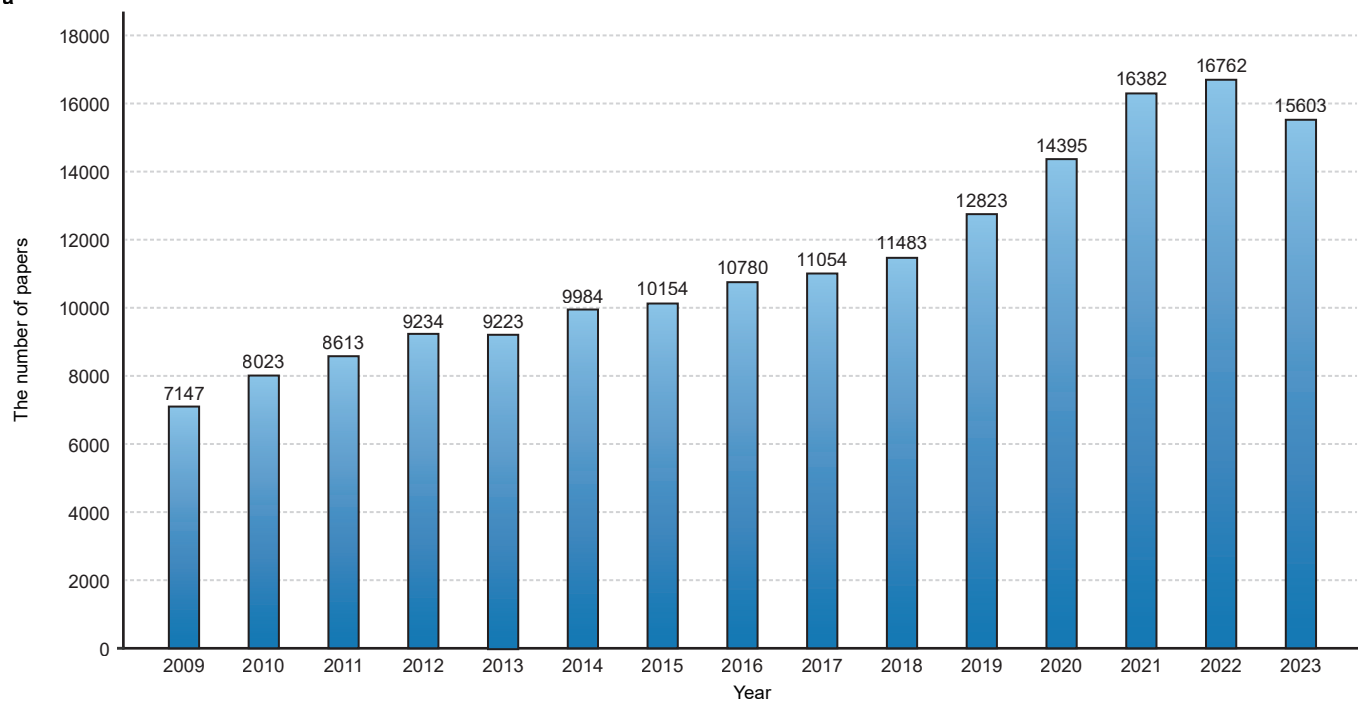

**b**

- Proteomics-related
- Disease-related

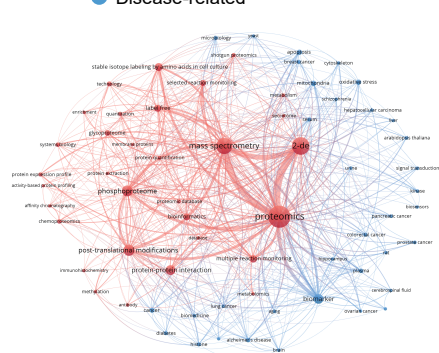

from 2009 to 2013

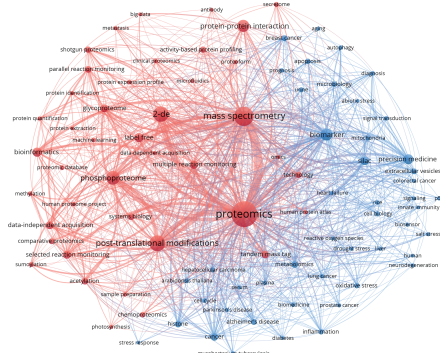

from 2014 to 2018

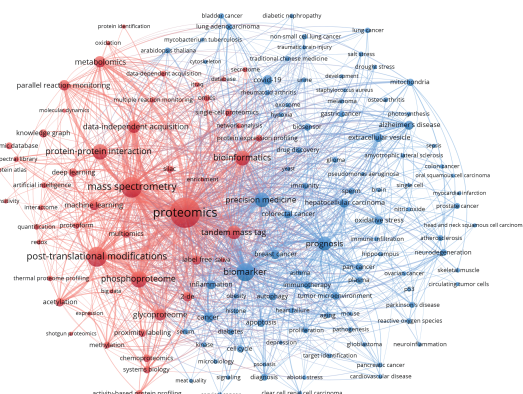

from 2019 to 2023

Supplement: nwaf278_Supplemental_Files [file nwaf278_supplemental_files.zip › Fig._S2.pdf]

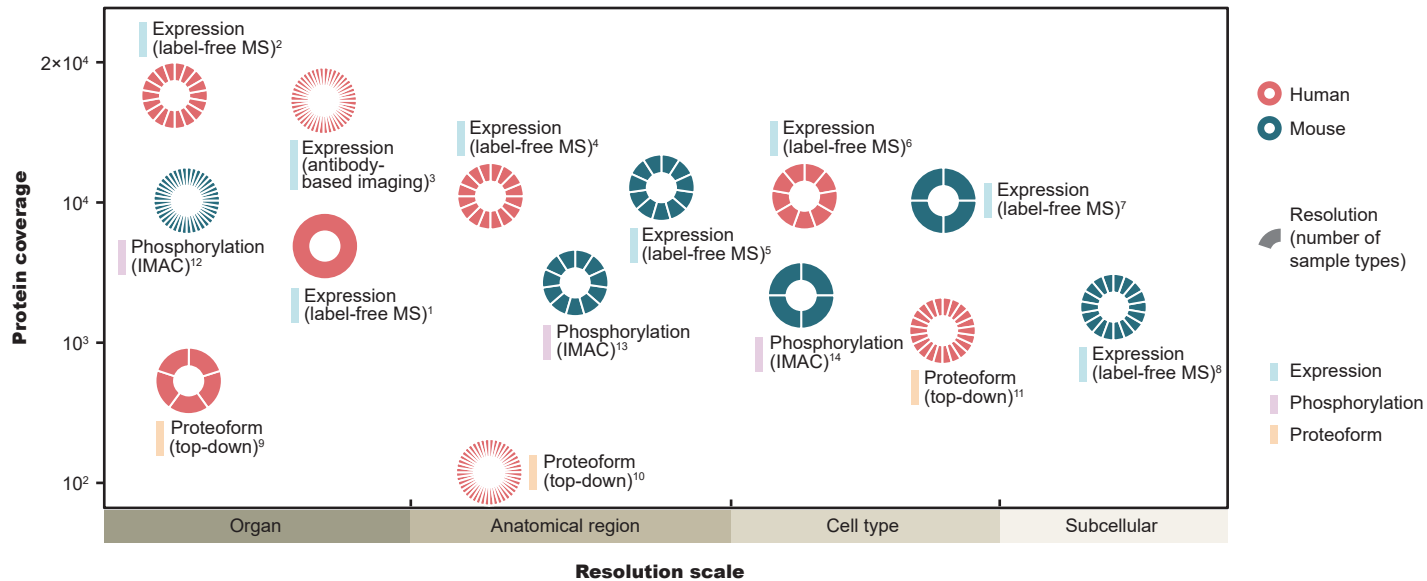

Supplement: nwaf278_Supplemental_Files [file nwaf278_supplemental_files.zip › Fig._S3.pdf]

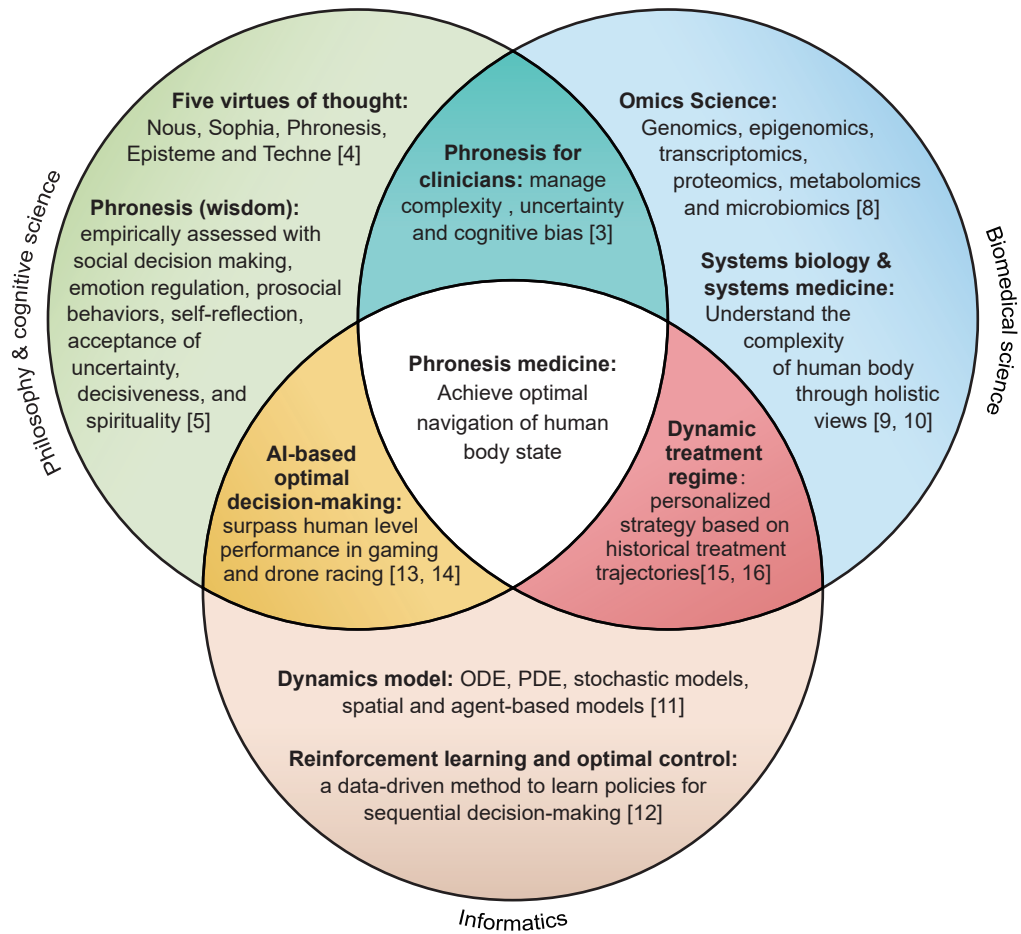

Supplement: nwaf278_Supplemental_Files [file nwaf278_supplemental_files.zip › Fig._S4.pdf]
